# Defining collective irrationality of COVID-19: shared mentality, mimicry, affective contagion, and psychosocial adaptivity

**DOI:** 10.3389/fpsyg.2023.1192041

**Published:** 2023-07-06

**Authors:** Wojciech Kaftanski

**Affiliations:** Human Flourishing Program, Harvard University, Cambridge, MA, United States

**Keywords:** collective irrationality, COVID-19, affective contagion, weak mentality, psychosocial adaptation, mimicry, adaptive strategy, collectivity

## Abstract

This paper defines the nature of collective irrationality that flourished during the COVID-19 pandemic and lays out specific individual and shared traits and dispositions that facilitate it. Drawing on the example of globally experienced phenomenon of panicked toilet paper buying and hoarding during the COVID-19 pandemic and resources from philosophy, psychology, sociology, and economics this paper identifies four essential features of collective irrationality: weak shared mentality; non-cognitive and immediate mimicry; affective contagion; and psychosocial adaptivity. After (I) initially pointing out conceptual problems around benchmarking collectivity and irrationality, this paper (II) identifies weak mentality as serving the goals of “group” recognition internally and externally. It is argued that (III) the non-cognitive and immediate mimicry and emotional contagion are shared and individual dispositional conditions that facilitate collective irrationality in environments affected by uncertainty (IV). The human mimetic faculty and susceptibility to emotional contagion are presented as enabling and augmenting conditions under which collective irrationality flourishes. Finally, (IV) presenting collective irrationality in the context of psychosocial adaptivity, the paper provides evolutionary reasons for engaging in irrational behaviors, rendering collective irrationality as an adaptive strategy.

## 1. Introduction

The COVID-19 pandemic has been accompanied by an unprecedented occurrence of panic purchasing and amassing of goods worldwide (Chua et al., 2021). One particular good has become an unexpectedly invaluable commodity during the pandemic: toilet paper. This globally experienced phenomenon of panic buying and hoarding toilet paper baffled both the doers and the observers alike, and it rightly became of interest to the academic community (Bentall et al., 2021; Leung et al., 2021; Taylor, 2021). While this phenomenon has been linked to a feeling of perceived scarcity, grounds for the emergence and proliferation of this perception of scarcity have not been established. Furthermore, scholars have been mystified by the commonplace connection between toilet paper and the COVID-19 pandemic. Indeed, there is a lack of a direct or implied causal relation between toilet paper and known COVID-19 symptoms in public health. This negative association between known COVID-19 symptoms and stockpiling of toilet tissue is considered here a prime example of human collective irrationality (*CI*) meaning actions taken that are both irrelevant to and not contributing to (not advancing) one’s wellbeing. This contrasts with actions that have some recognizable degree of furthering one’s best interest, at times against the interest of others, such as for instance stockpiling of COVID-19 medicines and vaccines causing disparity in national or regional distributions and supply chain-crash; (Asundi et al., 2021).This is also in contrast to acting against one’s best interest by disregarding one’s better judgment or values (Szanto, 2017).

While not the only example of collective irrational behaviors (*CIs –* collective irrationalities in a general sense), the phenomenon of people panic buying and hoarding toilet paper is a prime example of an irrational collective (*CI*) behavior that operates irrespective of the principles of logic or probability fundamentally attributed to informed decision making. Taking the phenomenon of panicked toilet paper buying and hoarding as exemplary of collective irrationality (*CI*) that emerged during the pandemic, the aim of this article is to define the nature of this irrational collective behavior and lay out specific individual and shared traits and dispositions that facilitate them. To achieve its goal, this paper consults sources from philosophy, sociology, psychology, and economics to present a more wholesome account of *CI*. This approach to *CI* provides a broader and more coherent account of the “nature” of *CI* across these disciplines, which, on their own, tend to focus on one particular aspect of *CI*. It also expands our knowledge of the complexity of collective irrational (*CIs*) behaviors in a more general sense.

The article has four key parts. The first part (I) offers an introductory reflection on the key definitions that are fundamental to *CI* such as collectivity and individuality, and irrationality and rationality. It draws attention to the complex nature of irrationality and collectivity, and indicates the conceptual problems around benchmarking irrationality; it also argues that collectivity should not be understood as tantamount to an aggregate of individuals. The second part (II) considers *CI* in relation to shared mentality. It argues that *CI* is characterized by a shared mental content understood in a weak sense (“weak mentality” or “limited mentality”). The third part (III) considers mimicry and affective contagion as dispositional conditions that facilitate *CI* in environments affected by uncertainty. It is argued that the human mimetic faculty and susceptibility to emotional contagion enable and augment conditions under which *CI* flourishes. In the penultimate part (IV), *CI* is rendered in the context of psychosocial adaptivity, explaining evolutionary reasons for engaging in irrational behaviors, presenting *CI* as an adaptive strategy.

## 2. Preliminary definitions

### 2.1. Defining collectivity

Collective irrationalities (*CIs*) pose conceptual headaches from the start. What does it mean for something to be collective? Likewise, when does something qualify as irrational? Collectivity is problematic to define. Does it come in degrees? Collectivity may mean an assemblage of objects—in contrast to a single object—that are somehow similar and are in a “relevant” proximity. However, not all aggregates of similar objects must necessarily be understood as collectives. In nuclear structure studies we observe non-collectives, which despite shared similarity and proximity, do not interact with each other (Knežević et al., 2022). Is the world we encounter one large collective, or perhaps a collective of collectives? Brain is an example of a collective object; it is an aggregate of spatially distributed neurons that gather and process data that, ideally, produce “coherent behaviour at the whole organism level”(Daniels et al., 2016). Neurons in the brain are not centralized but are distributed across various areas responsible for different cognitive and motoric functions (Zhang et al., 2016). While impaired cognitive-motoric functions are often diagnosed as caused by asynchronous or uncoordinated interactions between neurons, not all concerned neurons act in a synchronous and coordinated manner in non-pathological settings. Moreover, while not all neurons are engaged in all processes in the brain—even when they belong to a particular brain area responsible for given functioning—they are generally considered to be part of the collective we call the brain.

Space and proximity are characteristics that help us describe collectivity (Lee, 2017; Wang et al., 2021). Spatiality is a key factor at work in defining the formation and functioning of various social networking (Bosco, 2001). One may observe that, in a certain space, there is more than one person out there. As it would be arguably problematic to include elements outside the brain to count as the elements of the brain, it would be analogously problematic to assume collectivity when addressing people spread across an immensely vast area. However, space and proximity do not guarantee collectivity. Arguably, the advent of the Internet, but also preceding it the phenomenon of newspaper readership have contributed to the formation of forms of virtual and dispersed collectives. However, such *CIs* are not as I argue eminent of the collective phenomena that are COVID-19 specific—we have witnessed them in the pro-COVID-19 times. Moreover, there is no consensus in the scholarship on whether such dispersed phenomena as echo chambers, epistemic bubbles, and conspiracy theories should be perceived as forms of *CIs* (*Cf*: Coady, 2007; Brotherton et al., 2013; Lukić et al., 2019; Nguyen, 2020).

Do the passengers on a bus or customers in a mall constitute a collectivity, then? It seems that, apart from a form of proximity, collectivity requires a mental component via which individuals perceive themselves and are recognized as forming a collective entity. Robust forms of collectivity require a relatively robust mental content. Such mental objects speak about the nature of agency in groups. Pettit (2003) lists four types of groups, such as “collections,” “cooperatives,” “unified cooperatives,” and “the self-unifying cooperatives,” determining that only the last one is capable of agency and akrasia. While not differentiating between them in a systematic manner, Pettit argues that collectives represent groupings that cannot have intentional attitudes in a serious, literal sense, just in virtue of most of its members having corresponding individual attitudes. The members must form intentions about what is to transpire, they must reveal those intentions to another, and they must adopt measures that give effect to relevant intentions: measures such as those involved in accepting a certain formula as a matter of joint belief or endorsing a certain authority of behalf of the group (Pettit, 2003, p. 72).

Such a high bar for group agency is topped by Pettit’s insistence that group intentionality is aligned with rationality discernible internally by group members and externally by observers. Unified cooperatives can also be considered intentional agents, even though, as it is in the example in “non-human animals like cats and dogs,” they cannot behave irrationally in the sense of acting against their instincts (Pettit, 2003, p. 77). There seems to be a large gap between these accounts of agency. Can one reconcile so conceptually refined form of cooperation in humans with non-human agency incapable of akrasia?

Tideman (2017) sees collectivity as being founded in an intentional act that transpires between two or more agents, and which aims at establishing a rapport between them. He defines collectivity as follows: “a set of conscious individuals who perceive themselves as separate beings and also perceive benefits that might be obtained by coordination among themselves” (Tideman, 2017). His definition of collectivity is less demanding than Pettit’s in terms of the conditions of mentality (*coordination* rather than *cooperation*) and the internally and externally recognizable rationality; yet it necessitates individuality, which is problematic for Pettit’s inclusion of unified cooperatives in group agents. Tideman’s definition considers non-human animals such as dogs and chimpanzees as collectivities, yet it excludes “a flock of birds.” While not a strong claim in Tideman (“probably…not”), his view assumes that, as per his definition, a flock of birds might not be able to perceive benefits that stem from the implied bonding. The phenomenon of bird swarming, especially observed in murmuration (a deliberate and coordinated anti-predatory behavior formation), seems to contradict the view that only collectives so defined can benefit from controlled collectivity. Indeed, it is the collectivity of the birds that guards them against predators (Harley, 2021). Pettit and Tideman’s views of, respectively, cooperation and coordination in collectivity do not openly consider the complexity of collective behaviors that can be so different as “cooperating and competing,” “borrowing and improving,” “differentiation and copying,” “monitoring and sanctioning,” and others, many of them engaged by such complex animals as ants (Goldstone and Gureckis, 2009).

Tideman’s examination of voting demonstrates that (1) voters do not have to engage robust mental objects when voting in a coordinated manner and (2) voters can vote for the same candidate for different if not opposing reasons. Contra Tideman, however, in my example of toilet paper buying and hoarding I argue that people can be perceived as collectives that engage in competitive (hence uncoordinated and non-reciprocal) actions, partially, because they subscribe to ensuing benefits that are non-reciprocal and exclusive.

### 2.2. Defining irrationality

It is perhaps helpful first to think about rationality in order to conceptualize irrationality. We are rational when we reason, calculate, consider, and use concepts, hence engage mental objects by our higher faculties of judgment and discernment (Knauff and Spohn, 2021). Throughout most of the history of philosophy, rationality has been viewed as being superior to either what does not measure up to its standards or that which intentionally situates itself in opposition to. Rationality carries a certain normativity. What is rational is or should be the norm in both the theoretical and the practical domains of reasoning. The former asks about what it is reasonable to think or believe; the latter asks about what is rational to do, intend or desire to do (Audi, 2004).

The threshold of rationality is difficult to determine. There must be reasons for something to be rational. The paradox of irrationality formulated by Davidson (2004, p. 185) warns that “if we explain [irrationality] too well, we turn it into a concealed form of rationality”. Stein’s (1996, p.4) *standard picture* of rationality stipulates that “to be rational is to reason in accordance with principles of reasoning that are based on rules of logic, probability theory and so forth”. The threshold of rationality invites two crucial questions from two angles: philosophical and psychological (Bortolotti and Miyazono, 2021, p. 18). The philosophical question asks about necessary/sufficient conditions for a person to be rational. The psychological question asks about whether humans “naturally” act rationally. Historically, irrationalism is associated with the primacy of instinct or feeling over and against reason. In comparison to their rational counterparts, irrational behaviors and dispositions are generally found to be strange and unexpected.

Irrationality and collectivity form an interesting duo. For many classical philosophers, such as Plato and Aristotle, individuality was considered a promising ground for a good life principally governed by reason. It was considered the foundation of a larger collective, such as the family or the state. Modern philosophers, such as John Stuart Mill and Adam Smith, assumed that rationality was at work in more complex manifestations of human collectivity, such as economic markets. And that these “system-level phenomena” could be understood through the operationality of “the aggregation of individual beliefs, desires, and predictions” within these environments (Huebner, 2013, p. 221). Whereas these philosophers believed that individuals are essentially influenced by the societies in which they live, the underlying conviction of that belief is that the societies themselves are, in the first place, formed by individuals. While it was accepted that individuals within a collective engage in behaviors contingent upon the behaviors of their fellow group members, it was believed that both the motivation for such engagements and the types of interaction between individuals were rational and practical (“utility-driven”) (Goldstone and Gureckis, 2009).

Two foundational thinkers in sociology, Gustave Le Bon and Gabriel Tarde, reversed the perspective on human individuality and irrationality indicating that individuality is strictly linked with, if not shaped by, collectivity, while irrationality is intrinsic to collectivity. This early twentieth-century assertion, as Borch (2006) observes, was largely ignored in the approach to the study of crowds in the Anglo-Saxon context, especially in North America. This “catching up” with Le Bon and Tarde in the humanities, coupled with empirical research in human behavior and neurosciences, resulted in a reconsideration of the role of traditionally understood human agency in collective behaviors. The common denominator in these complex discussions that often occur on the borders of distinct disciplines such as sociology, psychology, economics, but also social cognition, evolutionary biology, and neuroimaging is *imitation* (Heyes, 2001, 2021; Bandura, 2021). More specifically, it is argued that multiple processes that underwrite human interactions occur under our cognitive register and belong to our evolutionary toolkit that secures our survival.

The primary focus in this article is COVID-specific *CI*. *CI* is defined as denoting a type of behavior whose origination and substance is irreducible to the sum of individual actions and qualities that contribute to them. The emergence and the nature of *CI* cannot be ultimately causally traced to one or several individuals. Subsequently, the shape of *CI* cannot be predicted or modelled by analyzing individual factors. The nature of *CI*, but also the conditions under which *CI* emerges and flourishes, can be explained in relation to a number of concepts that have their roots in our evolutionary composition qualified by imitation. In context of the COVID-19 pandemic, especially, *CI* is an adaptive mechanism that is engaged when people are faced with heightened uncertainty that has the potential of morphing into panic. Not all *CIs* are COVID-19 specific, though. Focusing on *CI* that emerged during the pandemic of coronavirus, exemplified in toilet paper buying and hoarding, four essential features of *CI* are identified: weak shared mentality; non-cognitive and immediate mimicry; affective contagion; and psychosocial adaptivity. Moreover, *CI* thrives in environments that foster close proximity between individuals and in which individuals experience feelings of perceived uncertainty and scarcity.

## 3. Weak/limited shared mentality

There are different types of groupings. We attribute to them different properties. Social ontologists discuss such themes as group intentions, agency, reasoning, responsibilities, obligations, and blameworthiness, but also solidarity, cooperation, and wellbeing (*cf.* Collins, 2019). Focusing on mentality and mental states, one claim of this paper is that people groupings in which *CI* occur are identifiable as such internally and externally by their limited shared mentality. This is to say that *CI* does not need a robust mental component to occur and to be recognizable. It would be difficult to ultimately delineate between robust and weak mental objects. Customarily understood, the former pertains to a shared mentality that offers grounds for ascertaining collective responsibility and joint decision making. Hence, a robust mental object in group mentality centers around shared and informed intention to achieve a particular goal. Mental objects in a weak sense are produced, often without a specific intention, by spontaneous expression and internalization. In consequence, these mental objects are being shared without any structural planning; they also may be ambiguous or contradictory. What stands as a mental object in a limited or weak sense can be a belief about a situation or a set of circumstances that allow a group to be recognized as being about that belief, both internally and externally. Recognition of such a spontaneously conjured group mental object can be advantageous to the functioning of the group (in-group cooperation) or can help some in the group to outperform others (in-group competition) in the group. “The same motivations that lead to competition between companies, countries, or teams can lead to cooperation among the members of a single company, country, or team,” point out Goldstone and Gureckis in their “Collective Behavior” (2009).

Pondering the nature of group mentalities, Huebner states that “‘herd mentality’…is not mentality at all” (Huebner, 2013, p. 222). Herd mentality is thence essentially reducible to an aggregate of individual mental states. When we speak of a crowd being angry or getting angrier, we mean, according to Huebner, that individuals in the crowd are getting angrier. His reasoning is that “individual behavior within a herd is determined by the state of the local environment (including relative position to other animals), it is only necessary to posit individual states and processes that are sensitive to the behavior of others” (Huebner, 2013, p. 221–222).

Huebner’s view of herd mentality is a promising starting point for distinguishing collectivity that is especially prone to irrational behaviors from collectivity that offers a more robust resistance to them. Motivated by the avoidance of “the ontological extravagance of 19^th^-century claims about group minds,” Huebner commits to a mechanistic view of herding. Herding is essentially a complex occurrence of strategic movements that, while occurring on the collective level, consists of an aggregated grouping of individual mental states, which, in turn, result from individual processing of information coming from the movements on the edge and in the vicinity of the herd (Huebner, 2013). Herding behaviors are then largely devoid of a mental component. This lack of a mental component explains the proliferation of such *CI* as the panic buying and hoarding of toilet paper. Such processes are instigated and regulated by sensory receptivity of the human body to others. Huebner sees the absence of the mental component in the fact that these imitative behaviors are not unified. This is so even though herding may in fact appear as being coordinated. Rather, as he points out, such behaviors are instigated on emotional and visceral levels, which he reduces to the individual’s “sensitivity” to “a wide range of imitative strategies and unreflective tendencies to adhere to the norms that govern their community” (Huebner, 2013, p. 222).

In contrast to Huebner, this paper argues that *CI* phenomena are not in their origination and nature reducible to the sum of individual actions and qualities that contribute to them. The emergence and the nature of *CI* cannot be causally traced to one or a few individuals. In contrast to Huebner’s view that collective actions without shared mentality can be effectively explained as an aggregate of individual behaviors “*without remainder*,” it is argued that there is a *remainder* and that *it* tells us something important about the nature of *CI* and *CIs* in a more general sense. Moreover, while some irrational collective behaviors may be ultimately immediate and unreflective, not all *CIs* are devoid of a mental content. The fact that people grouped in a herding behavior recognize themselves and are being recognized suggests that there is mentality in herding, yet in a weak sense. Such we-recognition may be temporary and it may be contingent on proximity between members of the collective. Some herding behaviors are oriented around symbolic representations; these are types of mentality understood, again, in a weak sense.

The irrational overstocking that we observe in toilet paper overbuying results from panic that creates a paradoxical form of bonding. We see this paradoxical nature of bonding caused by panic in the fact that individually and communally discharged panic brings people together; yet, it does so in an antagonistic and competitive manner. The paradoxical dimension of panic is well captured by Gibbs in her influential “Panic! Affect Contagion, Mimesis and Suggestion in the Social Field” (2008):

Panic presents this paradox: on the one hand it shatters any *esprit de corps* because it produces a situation of “each one for him/herself,” while on the other hand it represents the greatest moment of sensory receptivity of the human body to others—for in it, sympathetic or affective contagion is at its height (2008, p. 133).

Paradoxicality of panic pertains to a peculiar undetermined interplay of opposite types of group interactions among individuals, such as cooperation and competition. “Bound together” in a resolve to fend for themselves in the belief that the authorities are not doing enough to address the COVID-19 situation, people compete against each other in seeking to gather the most resources. Borch-Jacobsen (1988, p. 167) calls it bonding “in the mode of a non-bond.” The rivalrous frenzy of that desire is easily detected by both perpetrators and observers. It would not be uncommon, or indeed counterintuitive, to say that the crowd getting angrier is an observable fact. Thus, when we see a crowd getting angrier, it is not simply that we see the individuals who comprise the crowd are getting angrier.

The irreducible force of the crowd is what unites individuals in a common craving to obtain objects, which, considering their scarcity, may result in violence. Such violence is caused not simply by the aggregate of individuals. It is the *remainder* that topples cars in protests in some cases; it is the *remainder* that marches in peace in others. In the case of *CI* exemplified in the panic buying and hoarding of toilet paper, the feeling of we-are-all-in-this-together coupled with everyman-for-himself is the paradoxical bond that forms a weak form of we-recognition.

A representational object that assembles individuals around it can be understood as a shared mental object in a weak sense. Apart from acting in relation to such figurative shared object in the process of forming a paradoxical bonding, the latter is formed by a communal, often irrational, valuing of that object. Such a shared object is a symbolic representation (Passinsky, 2020). This representation need not be a fully-fledged mental object that is part of an advanced cognitive process.^1^ The function of symbols for collectivity is significantly elaborated by such classical sociologists as, for instance, Durkheim (1912/2001). In this context, as a collective representation the symbol is not something that becomes *fixed* and materialized in general culture (Durkheim, 1912/2001; *cf.* Arppe, 2016, pp. 82–90), because the emergence of *CI* exemplified in overbuying is often local and short-lived. Even if short-lived, representational symbols are evidence of a collective representation that requires collectivity for its development and subsistence.

## 4. Collective and individual dispositions

### 4.1. Mimicry

The panic buying and hoarding of toilet paper is a herd mentality phenomenon that has its roots in mimicry (Loxton et al., 2020). There are two dominant approaches to understanding herd behavior. John Maynard Keynes (Keynes, 1930/1971) defines herding in macroeconomy as an informed behavior of following collective decisions made by other people in uncertain times. Herding takes place when individuals perceive themselves as deprived of some important information and when they see others as being in possession of them. This model of thinking about herd behavior assumes that people’s imitation of others is conscious and results from reasoning. Keynes’s macroeconomy builds on Mill’s *homo economicus*, an ideal market participant who behaves rationally in a self-interested way to maximize their satisfaction (Persky, 1995). It is assumed that anomalous behaviors result from errors in perception and information processing (McFadden, 1999).

This model of herding has been criticized for ignoring the input of “socio-psychological influences” that often go beyond the assumptions of the rational principle in decision making (Baddeley, 2010). Market economy is not simply governed by informed decisions. Situations in which people make purchases they regret, cannot validate or explain are quite common. Alchian (1950) argues that reasons for, but more specifically the motivation behind, irrational activities in the sphere of market economy are not to be found in reason-governed “profit maximization.” Rather we should think about such behaviors as *explainable* in light of “the principles of biological evolution and natural selection by interpreting the economic system as an adoptive mechanism which chooses among exploratory actions generated by the adaptive pursuit of ‘success’ and ‘profits’” (Alchian, 1950, p. 211). Alchian contrasts “profit maximization” with “positive profits,” indicating that the latter have an adaptive function and adaptive motivation to them. Especially, under a condition of “pervasiveness of uncertainty and incomplete information,” market agents tend to resort to “adaptive, imitative, and trial-and-error” strategies, which are often manifested in *CI*.

An alternative to the *homo economicus* model for herd behavior regarding *CI* is to be located in the view of herding as largely occurring below the cognitive register and having a reflexive, hence automated nature. Herding so understood results from mimicry, which is a type of reactive imitation that bypasses the cognitive apparatus. Reflexive, hence non-reflective mimicry pertains to an involuntary, automatic, and often spontaneous transmission of expressions and behaviors, states, feelings, and emotions between people. It ranges from such basic mechanisms as autotuning one’s tone of voice, mannerism of behavior, to the sharing of emotions, and incorporation of values. While transpiring unbeknownst to its partakers, reflexive mimicry significantly influences the functioning of the partakers. Indeed, as Duffy and Chartrand (2015, p. 112) indicate, “Although the mimicker and the mimicked are generally not aware of its occurrence, it powerfully affects both their relationship and interactions with others by facilitating affiliation.” We distinguish different types of mimicry: “facial mimicry”; “emotional mimicry”; “behavioral mimicry”; and “verbal mimicry” (Duffy and Chartrand, 2015). Yet, they are all interconnected.

A large body of literature portrays how mimicry operates between neonates and parents, especially mothers, but also strangers (Salvadori et al., 2021). This continues throughout the life span. Surprisingly our tendency to imitation does not decline in adulthood (Keupp et al., 2018); indeed, some argue that it increases with maturation (McGuigan et al., 2011). It leads to overimitation, meaning faithful imitation of elements of the imitated action that are irrelevant to the intention behind it. Studies of overimitation in humans, which one would expect to diminish with adulthood with respect to reliance on the cognitive apparatus, hence selectivity, indicate that we incorporate tendencies from our near and distant environments to learn about how things work and what the social norms are (Kenward et al., 2011). It is important to note that these norms do not have be “reasonable” and they do not need to be permanent.

So understood reflexive mimicry “explains” such *CI* as panic buying and hoarding toilet paper. It transpires in close proximity when the mimicked and the mimicker can see each other. It is spurred and solidified by the condition of similarity. “The observer’s perception of the model’s behavior causes similar behavior in the observer, in some way such that the similarity between the model’s behavior and that of the observer plays a role, though not necessarily on the conscious level, in generating the observer’s behavior” (Hurley and Chater, 2005, p. 2). Hence, this urge to purchase and stockpile toilet paper plays on at least two levels. On the one hand, it is automated by the observance of behavioral patterns in others. In that sense it occurs below the cognitive register akin to a form of synchronization. On the other hand, there is agency and selectivity at work, which prompts the observer to attribute value to goods in specific contexts. In both cases the mimicker expresses situational norm-learning. As Chartrand et al. (2005) indicate, reflexive mimicry is based on the link between perception and behavior, but it is also augmented by a recognition of “affiliated goals” of the appropriated action. In the *CI* of toilet paper hoarding, the mimicker responds to a collective action recognizing the localized set of norms transpiring in such *CI*. His partaking in the phenomenon expresses that he subscribes to these localized norms and values. This normativization and valuation of *CI* is sanctioned from “the inside” of crowds; it is eventuated by cognitive and non-cognitive internalization and subsequent externalization of given norms and values in the particular framework by cognitive and non-cognitive imitation. Hence the normativization and valuation may come across as unreasonable and perhaps nonsensical to “outsiders,” and it may have a limited influence on them (“reduced spillover”).

### 4.2. Affective contagion

The case of panic buying and hoarding toilet paper shows that *CIs* are especially operative in environments that are significantly influenced by uncertainty (*cf.* Reiss, 1991; Sim et al., 2020; David et al., 2021). However, this does not mean that “uncertainty, and perceptions of scarcity” are the direct causes of “the panic purchasing behavior of consumers,” as indicated by the study of Omar et al. (2021). This paper argues that a proliferation of the perception of uncertainty has affective underpinnings. Conceptualizations of affect largely depend on the field of study; they are defined in relation to emotions and reasoning (and in some cases in relation to visceral factors). Some scholars understand affect as “the experience of *feeling* an emotion” where the latter is a biological response to external stimuli that involves “the recall and cognitive processing of affect” (Baddeley, 2010). One dominant view of emotions in philosophy attributes propositional attitudes to emotions indicating that they are in some respect expressions of our judgment about various objects in the world (*cf.* Thalberg, 1973; Solomon, 1977).

Debates on whether emotions necessarily have objects and whether they are intentional (Lamb, 1987; Price, 2006) have challenged the cognitive view of emotions, opening up a possibility of considering some emotion-like phenomena as being objectless, non-reflective, and embodied. Arguably, these three elements are constitutive of affect, following Massumi (1995, pp. 84-88).^2^ More specifically, affect is an action-generating feeling experienced in the body, independent from emotions and cognition (Kaftanski, 2021, pp. 171–172). This means that affects are often beyond our control. Affectivity is also privileged in terms of the length of time needed to generate a reaction. For instance, the arousal of fear in us in a hazardous situation is first processed and evaluated by the body, which generates the effect of hair standing on end, which is then translated into cognitively processed data that appraises the situation we are in. Affect is also self-reflexive and self-enhancing. Discharge of affect in the body “tend[s] to rearouse the same affect. This is the principle of contagion—the fear-arousing potential of fear, the anger-arousing potential of anger, the excitement of excitement, the joyousness of joy, the distressing quality of distress” (Tomkins, 1962, p. 81).

Attempting to understand the phenomenon of overbuying, some scholars distinguish between panic buying, which is associated with perceived scarcity, and hoarding as being associated with “a general intolerance of uncertainty” (David et al., 2021). Yet this conceptual distinction does not explain the mechanism behind the unprecedented proliferation of perceived scarcity and intolerance of uncertainty in relation to toilet paper in the context of the COVID-19 pandemic. Indeed, a degree of scarcity of some objects on the market is not unprecedented (see for example the current situation with broken supply chains with auto parts; *cf.* White House, 2021), yet it does not cause panic buying of these or related objects in the sense presented here.

Trends in financial markets are often sourced from localized environments in political economy. For instance, it has been shown that the risk of contagion is much more present on the regional level than on the global level (Hedström et al., 2020). This localization of contagion (“reduced spillover”) indicates that our strategies in coping with uncertainty are localized. In situations marked by uncertainty, people tend to outsource decision making by committing to bypassing strategies that shorten the time needed to generate informed reactions. This “shortcutting” is part of our social learning—“reasoning the fast and frugal way” (Gigerenzer and Goldstein, 1996). Affective imitation can serve as a “fast and frugal heuristic” in social situations.

The key factors contributing to the *CI* exemplified in toilet paper buying and hoarding are the proximity between participants and observers, the reflexive nature of the imitation of behavior, the collective participation in this phenomenon by other people, and the affective contagion of the feeling of scarcity correlated with the stressor of uncertainty (Kannan and Koehler-geib, 2011; Karsai et al., 2014; Hanna et al., 2020) Affective contagion is a spontaneous and reflexive proliferation of feelings/affects in a group of people. Like mimicry, affective contagion is a process in which an observed behavioral change in one individual leads to the reflexive production of the same behavior in other individuals in close proximity. Yet, affective contagion has an element of motivational affectivity that is shared by both the observer and the mimicker.

Affective contagion is self-enhancing, meaning the effects of the discharge of affective emotions enhances the experience and influence of these affects on one’s perception and action. Discharged, affective contagion transpires before it is observed and can be cognitively assessed. Additionally, the perception of shortage and uncertainty, hence factors indicating adverse circumstances that produce anxiety, hamper our ability to reflect on the experienced situation and to “control” affective discharges. Affectively transmitted, uncertainty and scarcity are or become hard to penetrate cognitively, hence understand and regulate.

Analyzing the origins of panic in relation to affective contagion, Gibbs (2008) points out that panic is indeed the result of prolonged stress and anxiety, which “fragments” the self, leading it to carry out random and frenzied behaviors (Gibbs, 2008, p. 131). Caused by affective contagion, panic is paradoxical. In one sense it is embodied, reflexive, and largely collective (on the one hand, “it represents the greatest moment of sensory receptivity of the human body to others”); on the other hand, it “shatters any *esprit de corps* because it produces a situation of ‘each one for him/herself’” (Gibbs, 2008, p. 131). Lastly, proliferated panic contributes to “the irrationality of crowds, their impulsivity and their tendency…to descend into disorder and violence” (2008, p. 133). The infamous “cutthroat battles over toilet paper” in Australia and the United States, among others, are perfect examples of such violent behavior.

## 5. Psychosocial adaptivity

The literature that explains the role of imitation in collective learning is vast. One important perspective that is often missed in philosophical observations on irrational emotions and irrational behaviors is that of efficiency or success. In philosophical discussions we find efficiency to be a derivative of utility correlated with cross-situational consistency that engages logic and probability. This means that something is efficient when it is rational, hence when it responds to universalized rules governed by what is logical and probable. It is assumed that what is efficient is retained and the inefficient becomes eliminated. This is what drives *homo economicus.* Evolutionary psychologists note that efficiency is not always consistent across various, if not all, scenarios. Certain actions may be seen as irrational at a universal level, but not so at a local level. This is to say that what appears irrational may have some kind of logic built into it. But, this paper argues, the predicate of rationality is secondary to what becomes optimal in the given, localized situation. Alchian (1950, p. 211) says: “where foresight is uncertain, ‘profit maximization’ is *meaningless* as a guide to specifiable action.”

The panic buying and hoarding of toilet paper may make us worse off generally (ridding ourselves of financial resources to purchase important commodities in demanding situations), but it may make us better off locally (stocking up on locally valued goods). Hence, some behaviors that cannot be justified universally are perceived as advantageous locally. Herding behaviors triggered by “irrational” factors have deeply evolutionary underpinnings that developed to be operative especially in localized environments. “Proximate mechanisms such as herding, when motivated by emotional responses that appear irrational and motivated by emotions, in fact are engaging evolutionarily old but highly conserved brain mechanisms which may be locally optimal but are not necessarily universally optimal,” writes Baddeley (2010, p. 286).

Our reliance on herding mechanisms can be strictly “instinctual” where “instinct” refers to adaptive abilities (epigenetical or others) that do not result from learning and experience (Gottlieb, 1997). An example of such practice would be the “unlearned” instinctual predisposition to group against predators and in response to misfortune. Yet, it seems that panic buying and hoarding has a “learned” element to it. It is an enacted recognition of emerging localized trends reflective of changing group dynamics. This adaptive mechanism allows one to follow a group of people in a herding behavior pursuing situational optimization, hence minimizing vulnerability.

Conformity is an important point of reference in relation to *CI*. Conformity is defined differently across philosophy, psychology, sociology, and economics (Coultas and van Leeuwen, 2015). Donald (2005) sees our synchronization with group norms and patterns in mimetically triggered conformity, which is both cognitive and non-cognitive. “Conformity, on all levels of overt behavior, is one of our signature traits, conferred by a universal mimetic tendency. We conform not only to the immediate patterns of our social group but also to the internalized ideals and archetypes of that group” (Donald, 2005, p. 300). Griffiths (1997, p. 57) lays out that position thus: “The evolutionary psychologist takes a phenomenon like a common but ‘irrational’ pattern of reasoning and argues that this behavior was selected for some advantage that it confers”. This conferred selective advantage may be intransitive, though. This means that reasons for its preference and activation can only be traced back to collective action within the group, not to individual preferences or dispositions, as would be argued, for instance, from the Bayesian point of view (Gelman et al., 2017; Williams, 2021). Consequently, inferring individual or aggregate actions from *CI* phenomena is deeply problematic.

While a behavior may occur as if expressing in-group internally consistent preferences that reassure and reinvigorate the behavior, time and non-confirming feedback may challenge the definition of the behavior’s success. Local outbursts of irrational behaviors are rather short-lived and tend to disperse when the imitative circuit in a given group setting is exhausted. Extended time allows for cognitive appraisal that may render the engaged action as being of limited utility. Exposure to non-participation in *CI* (non-confirming behaviors) allows for confronting feedback that, in some cases, prompts efficiency reappraisal.

Shamay-Tsoory et al. (2019), in their “Herding Brains: A Core Neural Mechanism for Social Alignment,” interrelate emotional contagion, social conformity, and synchronization, showing their neural underpinnings. Understood as key manifestations of social alignment, emotional (affective) contagion, synchronization (mimicry), and social conformity (compliance with implicit and explicit norms) form a closed feedback loop between group members. Taken individually, these manifestations of social alignment are open to evaluatory interventions that can penetrate the feedback loop and disrupt social alignment. Such disruptions may challenge the stability of groups by introducing information that question their values, principles, or goals. Yet, working in synergy, emotional contagion, synchronization, and social conformity reduce the probability of loop penetration by outside interventions and successfully preserve the composition of groups.

Additionally, it seems that the neural underpinning of emotional contagion, synchronization, and conformity makes each of them individually trigger others; “it is possible that engaging in one type of alignment (e.g., synchronized walking) would activate the shared alignment loop, thus activating the two other types of alignment (emotional contagion and conformity)” (Shamay-Tsoory et al., 2019, p. 177). What Shamay-Tsoory et al. call “the feedback-loop model of social alignment” includes a reward system that is activated when an alignment is achieved. This is to say that achieved alignment of emotional contagion, motor synchronization, and norms/pattern compliance “activates brain areas associated with reward, potentially related to the sense of satisfaction that occurs when people experience connectedness. Neuroimaging studies of reward processing have identified a set of regions comprising, among others, the ventral striatum (*VS*), ventromedial prefrontal cortex (vmPFC), and orbitofrontal cortex (OFC)” (Shamay-Tsoory et al., 2019, p. 179).

The above formulated insights from evolutionary psychology and cognitive sciences illuminate the psychosocial adaptive function of *CI* on both the macro and the micro levels. *CI* as exemplified in panic buying and hoarding transpire when a set of mutually validating and enhancing conditions are met, protecting the continuance of such behaviors that are collective and irrational. The neural underpinning of *CI* found in emotional contagion, social conformity, and synchronization contribute to the fossilization of normativity in behaviors on a pre-reflective level, largely obstructing cognitive penetration. Yet the time factor, which essentially is reflection-generative, shows that *CIs* are very often short-lived. Some adaptive strategies may be ruled as ineffective and essentially abandoned on account of better ones.

## 6. Conclusion

While mostly beneficial locally, short-lived, and eventually penetrable by reasoning, *CI* can be detrimental to individual and communal wellbeing. Human proneness to affective contagion negatively affects our ability to use critical skills, detect self-deception, engage emotion-regulation, hence normalize the dynamics of *CI*. As a type of *CIs, CI* can boost the dissemination and adoption of beliefs that are blatantly false, hence contribute to the spread of conspiracy theories, forms of extremism, and fake news. Arguably, *CI* can lead to political unrest, market destabilization, and physical violence. Whether humans should intervene in response to *CI* is a matter of debate (paternalism, nudging, etc.). Yet to understand how to do so effectively we should first understand the nature of the subject in question and what contributes to its germination and subsistence.

The point of this article has been precisely to aid our understanding to this end. Insights from philosophy, sociology, psychology, and economics were engaged to offer a coherent picture of the nature and the conditions that facilitate and sustain *CI*. As a prime example of *CI* was utilized the well-known and widely experienced phenomenon of the panic buying and hoarding of toilet paper. More specifically, it was argued that *CI* is a form of group mentality characterized by a shared mental object understood in a weak sense. Such an object can be symbolic. It allows for *CI* to be internally and externally recognized and identified. It was also argued that *CI* is localized and short-lived in nature. Particular individual and shared attributes and dispositions that facilitate *CI* have been identified. They include our mimetic toolkit and proneness to affective contagion, both of which render *CI* as operative largely below the cognitive register especially in environments of close proximity. Lastly, this paper argued that *CI and CIs more broadly* should be seen as strategies belonging to our “environmental adoption” that have largely psychosocial but also neural bases, utilized in times of perceived uncertainty and scarcity.

## Author contributions

The author confirms being the sole contributor of this work and has approved it for publication.

## Conflict of interest

The author declares that the research was conducted in the absence of any commercial or financial relationships that could be construed as a potential conflict of interest.

## Publisher’s note

All claims expressed in this article are solely those of the authors and do not necessarily represent those of their affiliated organizations, or those of the publisher, the editors and the reviewers. Any product that may be evaluated in this article, or claim that may be made by its manufacturer, is not guaranteed or endorsed by the publisher.

## Footnotes

^1^While rationality is about and involves mental objects, it does not follow that mental objects require or only involve rationality. There seems to be a certain bias at work in considering mental objects in relation to collective intentionality by Huebner. Many definitions of reasoning argue that reasoning is about mental objects (Knauff and Spohn, 2021).

^2^The view of affects (and affectivity) as distinct and independent from emotions and cognition as presented by B. Massumi and other scholars such as W. E. Connolly has been critically challenged by R. Leys for drawing a link of distinction that, while argued at length in critical theory and other relevant literature, lacks confirmation in empirical studies; *cf.* (Leys, 2011).
